# *Mitragyna speciosa*: Clinical, Toxicological Aspects and Analysis in Biological and Non-Biological Samples

**DOI:** 10.3390/medicines6010035

**Published:** 2019-03-04

**Authors:** Vânia Meireles, Tiago Rosado, Mário Barroso, Sofia Soares, Joana Gonçalves, Ângelo Luís, Débora Caramelo, Ana Y. Simão, Nicolás Fernández, Ana Paula Duarte, Eugenia Gallardo

**Affiliations:** 1 Centro de Investigação em Ciências da Saúde, Faculdade de Ciências da Saúde da Universidade da Beira Interior (CICS-UBI), 6200-506 Covilhã, Portugal; vaniaandbia@hotmail.com (V.M.); tiagorosadofful@hotmail.com (T.R.); sofia_soares_26@hotmail.com (S.S.); janitagoncalves@hotmail.com (J.G.); afluis27@gmail.com (Â.L.); deboracaramela50@gmail.com (D.C.); anaaysa95@gmail.com (A.Y.S.); apduarte@fcsaude.ubi.pt (A.P.D.); 2 Serviço de Química e Toxicologia Forenses, Instituto de Medicina Legal e Ciências Forenses—Delegação do Sul, 1169-201 Lisboa, Portugal; mario.j.barroso@inmlcf.mj.pt; 3 Universidad de Buenos Aires, Facultad de Farmacia y Bioquímica, Cátedra de Toxicología y Química Legal, Laboratorio de Asesoramiento Toxicológico Analítico (CENATOXA). Junín 956 7mo piso. Ciudad Autónoma de Buenos Aires (CABA), Buenos Aires C1113AAD, Argentina; nfernandez@ffyb.uba.ar

**Keywords:** *Mitragyna speciosa*, kratom, secondary metabolites, therapeutic uses, toxicology, analysis

## Abstract

The abuse of psychotropic substances is a well-known phenomenon, and many of them are usually associated with ancestral traditions and home remedies. This is the case of *Mitragyna speciosa* (kratom), a tropical tree used to improve work performance and to withstand great heat. According to several published studies, the main reasons for kratom consumption involve improving sexual performance and endurance, but also social and recreational uses for the feeling of happiness and euphoria; it is also used for medical purposes as a pain reliever, and in the treatment of diarrhea, fever, diabetes, and hypertension. However, this plant has gained more popularity amongst young people over the last years. Since it is available on the internet for purchase, its use is now widely as a drug of abuse, namely as a new psychoactive substance, being a cheaper alternative to opioids that does not require medical prescription in most countries. According to internet surveys by the European Monitoring Centre for Drugs and Drug Addiction in 2008 and 2011, kratom was one of the most widely supplied new psychoactive substances. The composition of kratom is complex; in fact, more than 40 different alkaloids have been identified in *Mitragyna speciosa* so far, the major constituent being mitragynine, which is exclusive to this plant. Besides mitragynine, alkaloids such as corynantheidine and 7-hydroxamitragynine also present pharmacological effects, a feature that may be attributed to the remaining constituents as well. The main goal of this review is not only to understand the origin, chemistry, consumption, and analytical methodologies for analysis and mechanism of action, but also the use of secondary metabolites of kratom as therapeutic drugs and the assessment of potential risks associated with its consumption, in order to aid health professionals, toxicologists, and police authorities in cases where this plant is present.

## 1. Introduction 

Several botanical products can be used for recreational purposes, and the effects include changes in mood, perception, behavior, and even in physiological parameters [1]. Although consumers usually have the wrong idea that they are completely safe and consumption does not involve any social or health risks, it is important to note that evidence of toxicity of such products has already been reported [2,3]. Commonly, the consumption of certain plants is associated with specific locations where people use them for what it seems to be the “natural effects” of the plant compounds. Regardless, a recurrent problem is the purification of the natural compounds in order to achieve stronger effects. This justifies the importance of early studies on addiction potential and classification of newly emerging psychoactive compounds.

Globally, the figures concerning consumption of new psychoactive substances (NPS) of natural origin are not clear. According to the European Monitoring Centre for Drugs and Drug Addiction (EMCDDA), the proportion of seizures in the category of other NPS (including those of natural origin) was of 12% in 2013 [4].

*Mitragyna speciosa*, belonging to the *Rubiaceae* family, found in both Asia and Africa, is a good example of such a NPS of natural origin. It is known as kratom, kakuam, kraton, ketum, ithang, or thom in Thailand and biak-biak in Malaysia or krypton when combined with *O*-demethyltramadol [5,6,7,8,9].

In Thailand, kratom’s tree can mostly be found in the south of the country, and it is easily obtained from teashops, being used as a substitute for alcohol and opium.

Two types of kratom can be identified based on the color of the leaf vein, which can be either green or red. Locals usually prefer the red vein, characterized for its bitterness and longer effects [5]. Fresh leaves, at a dosage of normally 10 to 30 fresh leaves per day, are mostly used chewed swallowed as a powder, but they can also be dried for smoking or used to make tea [10].

In 1943, kratom was put under regulatory control by the Kratom Act in Thailand which was believed to be an economic move rather than a decision based on the concern for public health. During that time, taxes were involved with the opium trade and, because it was so expensive, people started to use kratom as a substitute, and this had consequences for the Thai government’s income. Later in 1979, kratom was classified in Category V of a narcotics classification by the Thai government in the Narcotics Act, the same as cannabis, opium, and hallucinogenic mushrooms (the least restrictive and punitive level) [10,11,12].

Kratom was originally used mainly for its medicinal value in treating mild medical problems, namely fever, diarrhea, diabetes, pain, as a wound poultice, and to reduce the strain and fatigue of physical labor; however, it became also known and used to suppress opiate withdrawal symptoms given its affordability and availability [11,12,13,14]. In recent years, young consumers started to use kratom tea as a base for a cocktail known as “4 × 100”, consisting of kratom tea, cough syrup, Coca-Cola, and ice cubes. This eventually became a concern since these consumers were using additives such as benzodiazepines to enhance the effects [10].

Vicknasingam et al. [13] studied the major reasons for kratom consumption and also the socio-demographic characteristics of its users. The study was conducted on 136 active users, of which 76.5% had a previous history of drug use. The presented reasons for kratom’s consumption were reduction in addiction to other drugs, improvement of opiate addiction withdrawal symptoms, and its affordability relatively to heroin. A lot of short and long-term users claimed to have felt an increase in their capability for hard work, activeness, and heightened sexual desire [13,15]. An anonymous cross-sectional online survey was conducted in the USA in 2006, where 8049 users were studied through available social media and online resources from the American Kratom Association [16]. This study concluded that kratom is primarily used by middle-aged (31–50 years) and middle-income ($35,000 and above) individuals, the main purposes being to treat pain (68%) and emotional or mental conditions (66%).

*Mitragyna speciosa* can still be easily found on the internet for purchase, being a rather cheap alternative for opioids that does not require medical prescription [17,18]. In Europe, products labeled as *Mitragyna speciosa* (‘kratom acetate’ or ‘mitragynine acetate’) have been available since the early 2000s [19]. In recent years, products containing kratom are sold as ‘incense’ for their psychoactive effects, but concentrations of these active components vary depending on the variety of kratom used, circumstances, and harvesting time. The United Nations Office on Drugs and Crime questionnaire on NPS also revealed that kratom was one of the top three plant-based substances, along with khat and *Salvia divinorum*. Because kratom was not often under surveillance in national drug abuse surveys, information on its prevalence has been limited. Kratom and its active alkaloids are not listed under the 1961 and 1971 Conventions, but several countries have made policies for its control, also including mitragynine and 7-hydroxymitragynine (7-HMG) [20]. According to the internet surveys by EMCDDA in 2008 and 2011, kratom was one of the most widely supplied NPS [21]. Currently, *Mitragyna speciosa* is not illegal in most European countries or in the USA. In many EU countries, such as Denmark, Latvia, Lithuania, Poland, Romania, and Sweden, *Mitragyna speciosa* and/or mitragynine and 7-HMG are controlled drugs due to their high misuse potential. In other countries they are under control by the narcotic laws, including Australia, Malaysia, Myanmar, and Thailand (which has legalized the use of kratom and cannabis plants for medicinal use on December 2018). In New Zealand, *Mitragyna speciosa* and mitragynine are controlled under the Medicines Amendment Regulations [21].

**Figure 1:**
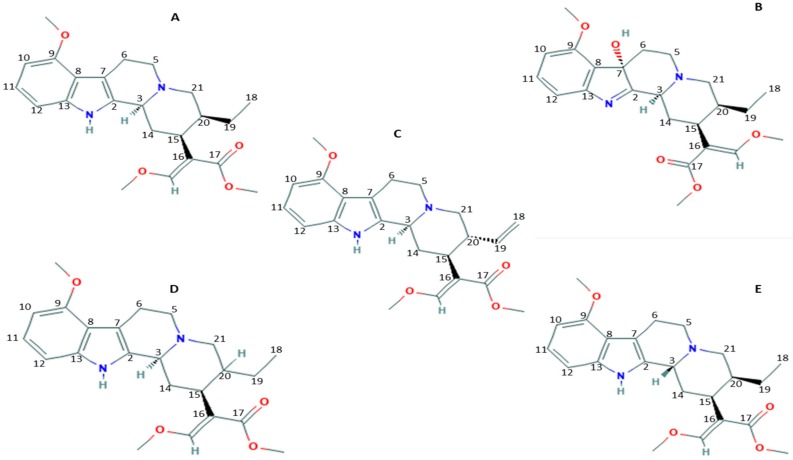
Structures of secondary metabolites: (**A**) mitragynine, (**B**) 7-hydroxymitragynine (7-HMG), (**C**) paynantheine (PAY), (**D**) speciogynine (SG) and (**E**) speciociliatine (SC).

Different formulations are available, including raw leaves, capsules, tablets, powder, and concentrated extracts. So far, more than 40 alkaloids have been identified in *Mitragyna speciosa*, the major constituent being mitragynine, which is exclusive to this plant [5,22,23,24]. Their relative amount varies monthly and according to the geographic origin of the plant [25,26]. Other constituents are paynantheine (PAY)–9%, speciogynine (SG)–7%, 7-HMG–2%, and speciociliatine (SC)–1% [25,27]. 7-HMG is a 7-hydroxyindolenine derivative of mitragynine (Figure 1) [28].

These are indole alkaloids of the corynanthe-type with a monoterpene (iridoid) moiety. Other compounds include raubasine and a few yohimbe alkaloids [26,29]. In general, kratom contains at least one alkaloid that can block calcium channels and reduces *N*-methyl-D-aspartate (NMDA)-induced currents [30]. Other compounds, such as flavonoids, terpenoid saponins, polyphenols, and various glycosides are also present [25]. Veeramohan et al. [31] performed a metabolomics study using the mature leaves of the green variety of *Mitragyna speciosa* in order to obtain a more complete profile of kratom’s secondary metabolites.

The alkaloids known to have a pharmacological effect are mitragynine, corynantheidine, and 7-HMG, but the remaining constituents might also provide this effect [25].

## 2. Research Methodology

The search for this review was conducted online on Pubmed, Google Scholar, and European Monitoring Centre for Drugs and Drug Addiction websites. Research papers, bibliographic reviews and case reports were included, the research done in Portuguese and in English. The search strings used were: “*Mitragyna speciosa*” and “consumption” and “toxicology” and “pharmacokinetics” and “case reports” and “pharmacological effects”. The search was performed between December of 2018 and January of 2019. No publishing date restrictions were used. In order to assess their relevance, all papers fulfilling the search strings were screened independently by four of the authors. Only those that were selected by at least two authors were subjected to review and were included in the manuscript.

## 3. Toxicokinetics and Pharmacodynamics 

Kratom’s pharmacokinetics in humans has not been well studied so far, and several factors such as metabolic half-life, protein binding properties, elimination rates, and metabolism are not yet known [26,32]. Studies in rats showed that the absorption of *Mitragyna speciosa* after oral administration presents a much smaller AUC compared to intravenous administration, despite the oral dose being higher. The low oral bioavailability may be related to poor aqueous solubility of *Mitragyna speciosa*, which results in a smaller fraction for absorption [33]. Mitragynine is also believed to be a basic drug, becoming highly solubilized and ionized in the stomach, which reduces its absorption and therefore bioavailability [34]. Furthermore, using Caco-2 cells to predict intestinal absorption, mitragynine showed better permeability than 7-HMG [35].

In terms of elimination/half-life, mitragynine demonstrated biphasic elimination from plasma, suggesting distribution into inner tissue compartments. However, given the short half-life (mean half-life of 2.9 ± 2.1 h after injection), it is rapidly eliminated. Still, there are some contradictory reports on this matter, stating a higher half-life for mitragynine [35,36]. A case report of a young kratom user showed that approximately 10–14 days after consumption has stopped kratom metabolites could still be detected in urine. Saturation of enzymatic pathways or high plasma protein binding could account for this situation, but none was proven to be right [35].

Concerning distribution, according to a case report, after conducting high-throughput molecular screening of mitragynine activity at central nervous system receptors, it was shown that it is a mu- and kappa-opioid agonist [37]. Mitragynine and 7-HMG are transported by passive diffusion, presenting reflux ratios of 1 and 1.2 respectively [35]. Also, a complementary pharmacokinetics study was performed, in which the effects of a single intravenous dose of mitragynine (5 mg/kg, mitragynine hydrochloride) were compared to those of either a single oral dose (20 mg/kg, mitragynine hydrochloride), lyophilized kratom tea, or the organic fraction of the lyophilized kratom tea at an equivalent mitragynine dose of 20 mg/kg in rats. After intravenous administration, mitragynine exhibited a decrease in the concentration–time profile, indicating its fast distribution from the systemic circulation or central compartment to peripheral compartments [38]. Another study was performed by Yusof et al. [39], who have evaluated for the first time the rate and the extent of mitragynine and 7-HMG transport across the blood–brain barrier.

Kratom metabolism is mainly hepatic and there is some evidence that it can affect the metabolism and efficiency of other drugs via induction of drug-metabolizing enzymes such as CYP450s and UDP-glucuronosyl transferase (UGT) [40].

An assay evaluated the effect of *Mitragyna speciosa* alkaloid extract on CYP, and has found that kratom was responsible for CYP3A4, CYP2D6, and CYP2C9 inhibition [41], but unfortunately there are no studies that can help finding which particular kratom alkaloid is responsible for this inhibition. Kamble et al. [42] verified that CYP3A4 was mainly responsible for the metabolism, with minor contributions of CYP2D6 and CYP2C9. The same authors have described that mitragynine was extensively metabolized in liver microsomes primarily to *O*-demethylated and mono-oxidated metabolites. Due to these cytochrome-related genetic variations in humans, these enzymes will partly account for inter-individual differences in drug metabolism and toxicity [43].

Additionally, Philipp et al. [44] observed that isomeric compounds found in the kratom users’ urine were SG and its metabolites, which can be also used as markers for *Mitragyna speciosa* presence.

Renal excretion of mitragynine is not considered significant, and mitragynine is not expected to be prone to substantial postmortem redistribution [36,45].

There are several studies that evaluate the effects of *Mitragyna speciosa* on human recombinant CYP450 enzyme activities [41]. This leads to implications, especially when mitragynine is co-administered together with herbal or modern drugs which follow the same metabolic pathway, contributing to herb–drug interactions [46].

According to Hanapi et al. [46], mitragynine might inhibit cytochrome P450 enzyme activities, specifically CYP2D6, and the strongest inhibitory effect was observed on CYP2D6, with a half-maximal inhibitory concentration (IC50) value of 0.45 ± 0.33 mM, followed by CYP2C9 and CYP3A4 with IC50 values of 9.70 ± 4.80 and 41.32 ± 6.74 μM respectively. Similar results are presented by Kong et al. [41], with apparent IC50 values of 0.78 µg/mL and 0.636 µg/mL for CYP3A4 and CYP2D6, respectively. Cinosi et al. [47] corroborate the possibility of a drug interaction if mitragynine and 7-hydroxymitragynine are administered together with drugs that are P-glycoprotein substrates. The authors mention, however, that *Mitragyna speciosa* is unlikely to have any significant clinical effects on CYP3A4 activity, but on the other hand might inhibit CYP2D6. Moreover, Lim et al. [48] performed an in vitro evaluation of cytochrome P450 induction and of the inhibition potential of mitragynine, and found that this alkaloid induces mRNA and protein expression of CYP1A2 consistent with the increased CYP1A2 enzymatic activity. Nevertheless, this alkaloid appears as a weak CYP3A4 inducer at the transcriptional level and a weak CYP3A4 enzyme inhibitor, leading to the same conclusion of Cinosi et al. [47], that it is unlikely to cause any significant clinical effects on CYP3A4 activity.

In this sense, the overall opinion is that the concomitantly administered drugs that may modulate these CYP isoenzymes activities will lead to clinically significant drug–drug interactions. Such interactions may result in serious adverse drug reactions, especially regarding drugs with short therapeutic windows such as carbamazepine, theophylline, digoxin warfarin, and phenytoin [49].

According to a review made by Ulbricht et al. [50], *Mitragyna speciosa* is likely to be unsafe if used by patients with neurologic disorders or that are taking either neurologic agents, such as alcohol, sedatives, benzodiazepines, opioids, or opium-containing products, or stimulant substances, such as caffeine, caffeine-containing products, cocaine, yohimbine, or related compounds. Also, the co-administration with monoamine oxidase inhibitors (MAOIs) is not advised. Recently, a case report of a fatality in a 27-year-old man was described by Hughes [51], who found a toxic blood concentration of quetiapine in conjunction with mitragynine.

Concerning the interactions of *Mitragyna speciosa* herb and food, there are some reports on this matter. According to a previous systematic review [50], the concomitant use of MAOIs, ayahuasca (*Banisteriopsis caapi*), syrian rue (*Peganum harmala*), or passion flower (*Passiflora incarnata*) with *Mitragyna speciosa* may potentially cause serious reactions. Moreover, yohimbe (*Pausinystalia yohimbe*) combined with *Mitragyna speciosa* may cause overstimulation and increased blood pressure, as it also occurs with the concomitant use of caffeine [50].

There have been reports on the concomitant use of opioids with *Mitragyna speciosa* causing oversedation or potential respiratory depression [52,53]. *Mitragyna speciosa* has reportedly been used for centuries for its psychoactive properties, opium-like effects, and ability to treat opioid addiction and opioid withdrawal. Reports starting in the mid-1800s give an account of *Mitragyna speciosa* being used as an opium substitute [54].

## 4. Clinical Effects/Pharmacology

Kratom has been found to have addiction potential in animal models when mitragynine and 7-HMG were given orally for five days [55].

### 4.1. Analgesic Properties

Mitragynine has a high affinity to mu-opioid receptors [56,57,58]. These receptors mediate analgesia, respiratory depression, and euphoria. It has been shown that its antinociceptive activity is mostly mediated by the supraspinal mu- and delta-opioid receptor subtypes, therefore making it the alkaloid responsible for the analgesic activity of kratom [57,58,59]. This also corroborates the claim that kratom can be used as an opium substitute or to diminish opium addiction, reducing the pain from withdrawal symptoms. Its affinity for kappa-receptors is considerably lower [58]. However, Stolt et al. [60] concluded that it exhibits analgesic effects via kappa-receptors and showed as well depressant effects on locomotor activity via presynaptic dopamine effects. The reinforcing effects of 7-HMG are mediated in part by mu- and delta-opiate receptors [61].

It is possible to use kratom as an anesthetic, and Vermaire et al. [62] reported the first case of this application by a patient using kratom for chronic pain.

Stimulation of post-synaptic alpha-2 adrenergic receptors, and/or blockage stimulation of 5-HT2 receptors by *Mitragyna speciosa* is also suggested. It is theorized that the effectiveness of the methanolic extract on alleviating positive and negative symptoms of psychosis may be due to inhibition of D_2_ and 5-HT_2_ receptors [63,64].

7-HMG also demonstrated high opioid receptor potency. In terms of analgesic activity, both mitragynine and 7-HMG were found to be more potent than morphine (mitragynine is about 13 times more effective, while 7-HMG is four times more effective) [13,55]. However, in a more recent study, kratom powder was found to have less affinity for the mu-opioid receptor than morphine [65].

At a cellular level, mitragynine can inhibit neurotransmitter release by reversibly blocking neuronal Ca^2+^ channels. It is proposed by the authors that the decrease in neurotransmitters leads to inhibition of pain transduction [66]. Mitragynine also inhibited adenylyl cyclase in NG108-15 cells through opioid receptors.

Cardiotoxicity was observed by induction of potentially fatal ventricular tachyarrhythmia (Torsade de Pointes). Blockage of the human Ether-a-go-go-Related Gene (hERG) channel in the heart constitutes a major risk of cardiotoxicity, and it is believed that *Mitragyna speciosa* suppresses hERG-mediated K^+^ currents and prolongs action duration [67].

Opioid agonistic activities were studied with twitch contraction in guinea pig ileum induced by electrical stimulation. The crude extract inhibited the twitch contraction, which was reversed by naloxone. Also, each extracted alkaloid (7-HMG, mitragynine, SC, PAY, and SG) inhibited the electrically-induced twitch contraction in a concentration-dependent fashion [68].

Both methanolic and the alkaloid extract of *Mitragyna speciosa* leaves were found to prolong the latency of nociceptive response on heat-induced pain in the hot plate test in mice, but not in the tail-flick test [69]. However, when Sabetghadam et al. [70] performed a study comparing the antinociceptive effects of alkaloid (20 mg/kg), methanolic (200 mg/kg), and aqueous extracts (100–400 mg/kg), they concluded that they all prolonged the latency of nociceptive responses in both tests. These analgesic effects were blocked by naloxone, which suggests partial mediation by opioid-receptors, similar to what occurs with morphine. Returning to the tail-flick and hot-plate tests, 7-HMG was again demonstrated to have a more potent antinociceptive activity than morphine. Its higher lipophilicity apparently makes it easier to penetrate the blood–brain barrier [71].

### 4.2. Anti-Inflammatory Properties

The anti-inflammatory effects of *Mitragyna speciosa* have also been studied [56,72,73]. The cyclooxygenase isoforms, COX-1 and COX-2, are involved in the inflammatory pathway that catalyzes prostaglandin PGE2 formation, which is one of the strongest inflammatory mediators. Mitragynine is capable of inhibiting COX-2 mRNA and protein expression, and therefore inhibits PGE2 formation. At lower concentrations it did not affect COX-1 mRNA and protein expression, but caution is advised at higher doses [72]. Overall, authors suggest that the anti-inflammatory properties of *Mitragyna speciosa* may result from a combination of inhibition of pro-inflammatory mediator release and vascular permeability in addition to enhanced immunity, stimulation of tissue repair, and healing processes [73].

### 4.3. Gastrointestinal Effects

Kratom also seems to have gastrointestinal effects [56]. The in vivo effect of the methanolic extract of kratom leaves in the gastrointestinal tract of rats reduced defecation frequency and fecal weight in castor oil-induced diarrhea. A single dose of the extract resulted in intestinal transit reduction; however, further decreases were not observed with prolonged intake. Since pre-treatment with naloxone had no impact on defecation frequency, it is believed that kratom may affect other pathways besides opioid-receptors [74].

Centrally injected mitragynine into the fourth ventricle of anesthetized rats resulted in a dose-dependent inhibition of 2-deoxy-d-glucose-stimulated gastric acid secretion; however, this effect was reversed by naloxone, which indicates the involvement of opioid receptors [75]. Mitragynine injected into the lateral cerebroventricle had no influence in acid secretion. Its effects of anorexia and weight loss may be related to the direct inhibition of neurons in the lateral hypothalamus. As for 7-HMG, a subcutaneous injection on mice resulted in gastrointestinal transit inhibition [71].

*Mitragyna speciosa* both acute and chronic effects include reduction of food and water consumption, and the additionally gained weight had a tendency to be reduced [76].

A study on the modulation of the glucose transport system of L8 muscle cells demonstrated that kratom can increase the rate of glucose uptake as well as protein levels of glucose transport, corroborating anti-diabetic effects [77].

### 4.4. Anti-Depressant Activity

Furthermore, kratom has also shown anti-depressant activity [78]. The overproduction of corticosterone reflects a hyperactivity of the hypothalamic–pituitary–adrenal axis, which provides a depression indicator. By significantly reducing corticosterone concentration in mice exposed to the forced swim test and tail suspension tests, mitragynine seems to possess anti-depressant effects [79].

Ismail et al. [80] also discovered an impairment between spatial learning and memory processing during chronic administration of kratom, observing learning deficits similar to those induced by chronic morphine or Δ-9-tetrahydrocannabinol treatment. However, an investigation on the acute effects of *Mitragyna speciosa* extract and mitragynine on short-term memory and motor coordination in mice showed that neither of them had a significant effect [81]. In 2018, a study conducted in humans [82] corroborated precisely these results, and apparently high intakes of kratom juice (>3 glasses daily) did not impair motor, memory, attention, nor executive function of regular kratom users.

### 4.5. Antioxidant and Anti-Bacterial Properties

As for antioxidant and anti-bacterial properties, *Mitragyna speciosa* proved to have both [83]. Nevertheless, there is no sufficient evidence that supports the use of *Mitragyna speciosa* for clinical indications and this becomes even clearer due to the contradictory information existing on this matter.

## 5. Toxicology

The pharmacologic effects of kratom leaves and their constituents are dose-dependent. Low to moderate dosages (1 to 5 g) can offer light stimulant effects to help workers against fatigue, while moderate to high dosages (5 to 15 g) may have opioid-like effects [13]. However, kratom also presents stimulant effects at high dosages (>15 g) [25,84]. Anxiety, irritability, and enhanced aggression are described, and long-term high dose consumption has been related to several atypical effects [13]. Hyperpigmentation of the cheeks, tremor, anorexia, weight loss, and psychosis have been noted in individuals with long-term addiction [84]. Although the use of kratom in opioid withdrawal situations is discussed in the scientific literature, some authors [85,86,87,88] have examined the consequences of withdrawal from kratom. Withdrawal is highly uncomfortable for some users, and as such maintaining abstinence becomes difficult. In fact, clinicians need to be aware of withdrawal symptoms and implement a similar approach as for opioid withdrawal scenarios, with long-term maintenance to prevent relapse. Trakulsrichai et al. [36] performed a study on the pharmacokinetics of mitragynine in 10 male subjects using kratom tea, and no serious adverse effects were found. All subjects have reported developing tongue numbness after drinking the tea and an increase in blood pressure and heart rate. These last symptoms had a delayed onset of 8 hours after tea consumption, which was even later than T_max_ (0.83 ± 0.35 h), and as such further studies are required.

A recent study reported the death of rats after treatment with 200 mg/kg total alkaloid extract [89]. Other assays showed greater toxicity of the alkaloid extract when compared to the methanolic extract, with LD_50_ values of 173.20 mg/kg and 4.90 g/kg, respectively, in mice. In a 14-day period to evaluate acute toxicity, 100, 500, and 1000 mg/kg doses of standardized methanolic extract were administered to rats and did not affect spontaneous behavior, food and water consumption, absolute and relative organ weight, nor hematological parameters. It did however significantly increase blood pressure one hour after administration, and the highest dose of extract also induced acute severe hepatotoxicity and mild nephrotoxicity after single dose administration [90]. Sub-chronic high doses of *Mitragyna speciosa* have also been found to damage the kidneys and lungs, as emphysema, over-inflation of the alveoli, and an increase in serum creatinine and blood urea were observed [91]. Furthermore, both the plant extract and mitragynine showed cytotoxicity to human neuronal cells, but no genotoxicity in the mouse lymphoma gene mutation assay (Saidin). Also, no mutagenic effects were observed in a study performed using the Ames test [92]. The results concerning the benefits and toxicity of kratom are inconclusive; for instance, Fluyau et al. [93] have not determined if the biochemical benefits of the plant outweigh its toxicity and risks. On the contrary, it seems that the potential side effects outweigh the benefits, and severe and real health hazards can, insidiously, lead to death.

Cases involving multiple toxicity and fatal outcomes after mitragynine or kratom use have been reported, but the underlying causes remain unclear. Recently, in 2019, Rusli et al. [94] attempted to correlate the effects of mitragynine with glycoprotein-P, a multidrug transporter which modulates xenobiotic pharmacokinetics and plays a key role in the mediation of drug–drug interactions. Using biomolecular techniques, these authors concluded that mitragynine interacted with important residues at the nucleotide binding domain site of glycoprotein-P’s structure, but not with the residues from the substrate binding site. Therefore, mitragynine is likely to be a glycoprotein-P inhibitor in vitro but it is not a substrate. Hence, concurrent administration of mitragynine-containing kratom products with psychoactive drugs which are glycoprotein-P substrates may lead to toxicity, and this can be clinically significant.

## 6. Case Reports

Some case reports have also been published. In the following section the main case reports found in literature will be discussed, in order to better understand the toxic effects of this plant. A 64-year-old male, who regularly used kratom to self-medicate his chronic pain, was found by his wife unconscious and seizing. A urine concentration of mitragynine of 167 ± 15 ng/mL was detected [95].

Nine unintentional deaths were reported due to consumption of krypton. Both mitragynine (0.02 to 0.18 μg/g) and *O*-desmethyltramadol (0.4 to 4.3 μg/g) concentrations were determined in postmortem blood samples [96].

A 44-year-old subject with chronic abdominal pain started taking kratom after reading about it on the internet. The increase of dose intake and subsequent attempts to reduce failed due to experiencing withdrawal symptoms. The patient gained 60 pounds, became lethargic and developed myxedematous face. A severe primary hypothyroidism was diagnosed, and the authors have related it to *Mitragyna speciosa* due to the reduction of the thyroid gland response to thyroid-stimulating hormone [97].

Another case report of a 25-year-old man suggested that kratom had induced intrahepatic cholestasis, which was confirmed by liver biopsy. Mitragynine was detected in both urine and serum samples [98].

Furthermore, a 44-year-old man with a history of depression, alcohol dependence, and other substance misuse was admitted for kratom detoxification. Prior to his admission, the patient was consuming approximately 40 g of kratom divided into 4 doses over 24 h. The patient also experienced withdrawal symptoms, even though he was regularly consuming kratom, which suggests the short half-lives of the active substances in kratom and a dependence syndrome primarily via agonist activity at the opioid receptors [99].

Domingo et al. [100] also reported a 22-year-old male with a history of drug addiction who used to mix an unknown amount of herbal substances. Prior to his death he fell from a window before going to bed but did not attend the hospital. A very high blood concentration of mitragynine was detected; however, the cause of death was determined to be aspiration of chyme. It is believed that, although kratom was not the direct cause of death, it had an important role in it. High doses produce sedative effects, and postmortem examination confirmed a humerus fracture of the left arm. It seems that the pain was alleviated by mitragynine, explaining the lack of urgency of the subject to seek medical attention [100].

Kratom’s consumption in Southeast Asia is changing. A recent study performed by Singh et al. [101,102] based on self-reported information suggested that prolonged kratom use does not result in serious health risks nor impairs social functioning. Two recent trends have also emerged: the first involves the reported use of kratom to ease withdrawal from opioid dependence in rural settings, while the second is related to adulterated kratom cocktails being consumed by young people to induce euphoria in urban areas.

The presented evidences corroborate the fact that kratom is not only toxic but can also be lethal. The Food and Drug Administration [103] reported 44 deaths associated to kratom use, one of them involving mitragynine alone. These findings have prompted the institution to issue warning letters to numerous businesses that were illegally selling kratom. Nevertheless, it is not always clear which substances may be responsible for the effects, because in many situations kratom is not the only product being consumed, hence further studies are required on this matter.

## 7. Analytical Methodologies

To date, there are some techniques that have been suggested for the determination of *Mitragyna speciosa*, with special focus on mitragynine [104]. These techniques are imperative, not only for identification and quantification of plant components, but also for metabolic studies, and forensic and clinical toxicology. Therefore, it is important to achieve easy, inexpensive, and efficient ways to identify this plant or its components in biological specimens [104]. An increased focus must be given when kratom is present with other products or when there is adulteration of its chemical composition. Chromatographic methods seem to be the most commonly used, with high-performance liquid chromatography (HPLC) being the most popular [105]. Other methods include gas chromatography and liquid chromatography coupled to mass spectrometry (MS). Genetic methods have inclusively been used to identify the plant [9]. Some of the methods are reviewed in Table 1 (biological samples) and Table 2 (plant material).

Kowalczuk et al. [106] proposed a comprehensive authentication procedure that involves botanical analysis of leaf material and mitragynine identification by thin layer chromatography (TLC) and HPLC. Although microscopic analysis cannot be used by itself for kratom identification due to fragmentation of material and similarities between species, it can help in gathering information on the characteristic elements of the powdered material, especially because of the difficulty of dried plant material analysis due to incomplete removal of chlorophyll.

Lesiak et al. [32] proposed the identification of unique biomarkers for *Mitragyna speciosa* recurring to high resolution (HR)-DART-MS, a robust and fast way for data acquisition, without the need for sample pre-preparation steps. Fresh leaves and leaf extract analysis allowed the identification of *Mitragyna speciosa* following the detection of mitragynine isomers and 7-HMG. However, there was a significant difference in the amount of information provided by each different specimen, with a drop of 56% of the obtained information when the leaf extract was used. The possibility of identifying *Mitragyna speciosa* by comparison of the mass spectral data with those of unknown plant leaves was also approached with the help of linear discriminant analysis (LDA), being successful [32].

Parthasarathy et al. [107] reported a method with HPLC-diode array detection (DAD). DAD may be used for *Mitragyna speciosa* analysis because it stands as a fast and simple method, although it is not specific enough and seems to lack clear definition [105]. Comparison of extracts showed a higher mitragynine concentration in methanol and alkaloid-rich extracts when compared to the water extract. This is explained by the poor mitragynine solubility in water. Acid–base extraction technique also increased mitragynine concentration by converting the compound (and also other alkaloids) into its salt form, which is water soluble, and then back extracting the compound into the organic layer after neutralization, resulting in more concentrated extracts [107].

Ion mobility spectrometry (IMS) has also been used for the detection of mitragynine in 15 commercial samples. Its high sensitivity, at the ng level, and portability makes it attractive for screening. Fuenffinger et al. [108] used IMS and later compared the results with those obtained using liquid chromatography–tandem mass spectrometry (LC–MS/MS). LC–MS/MS detected mitragynine in one additional sample, whose concentration was below the IMS detection limit.

Furthermore, a monoclonal antibody (MAb) against mitragynine was produced and its ability to provide detection in leaf samples was also studied. The immunogens were prepared by means of the glutaraldehyde and carbodiimide methods. Immunogenicity was confirmed by determining the hapten numbers using matrix-assisted laser desorption/ionization time-of-flight mass spectrometry (MALDI-TOF-MS). This method provided a rapid and sensitive way of mitragynine quantification [109].

Gas chromatography (GC) systems are viable and readily accessible due to their high selectivity and relative low maintenance costs. Their coupling to MS provides good determination capabilities for compounds. Still, there are some issues that cannot be ignored. The high temperature required for alkaloid elution combined with the upper temperature limit of the polymeric GC stationary phases impairs the parametric adjustment of resolution of alkaloid mixtures, and derivatization is often deemed necessary [105]. In addition, these systems are not capable of adequately resolving a number of diastereomers, such as mitragynine’s. This stands as an even bigger issue given the importance of mitragynine in *Mitragyna speciosa* analysis [105]. A study using GC–MS for urine samples could only detect mitragynine metabolites and some SC, SG, and PAY metabolites, with detection limits of 100 ng/mL for all diastereomers and PAY [110].

A comparison between GC and MS, supercritical fluid chromatography (SFC) and DAD, and UHPLC and MS and DAD for the analysis of mitragynine and other indole and oxindole alkaloids reported the GC method as less satisfactory. It proved to be unable to resolve mitragynine and SC. These diastereomers are only differentiated by the orientation of a single inner hydrogen atom, which makes the separation very hard by GC with a liquid stationary phase. In addition, the use of standard capillary columns may also have contributed to the inadequate resolution of the compounds.

SFC methods stood as a better choice given that they are faster, simpler and do not require as much organic liquids as the HPLC techniques [105]. Both were able to analyze mitragynine and other indole and oxindole alkaloids, but they differ in their effectiveness to identify mitragynine when its stereoisomers SG and SC are also present; indeed, and although mitragynine is the main compound of *Mitragyna speciosa*, it is important to find methods capable of identifying possible interferences from other diastereomers, which highlights the need for chromatographic separation [105].

The extraction technique can influence the yield in raw extracts and the relative alkaloid content of *Mitragyna speciosa* leaves, also highlighting the importance of extraction optimization. Mudge et al. [111] used the HPLC–UV technique to determine mitragynine and 7-HMG, and compared extraction solvents reporting methanol as the best choice; furthermore, they observed better extraction efficiency of mitragynine with 70% methanol with 0.5 M acetic acid. Other factors, such as the extraction method (shaking versus sonicating), solvent volume (10 versus 20 mL), and time (30 versus 60 min) were evaluated. Although results were inconclusive, shaking seemed to improve precision [111]. Other assays [112] compared ultrasound assisted extraction (UAE), microwave-assisted extraction (MAE), and supercritical carbon dioxide extraction (SFE-CO_2_) (using methanol, ethanol, water, and binary mixtures). Using LC/ESI–MS analysis, MAE (methanol:water, 1:1) gave the highest alkaloid fraction amount, while UAE showed the best yield for mitragynine. The authors concluded that UAE (methanol:water) seemed to be the most effective method to obtain a large quantity of the alkaloid [112].

Scientific literature concerning the determination of the main active compounds of *Mitragyna speciosa* in biological fluids is very scarce, mostly focusing on urine specimens. In these cases, hydrolysis is usually performed with β-glucuronidase and/or arylsulfatase because metabolites are majorly excreted in the conjugate form [110,113,114,115,116]. Regarding extraction procedures, some authors use liquid–liquid extraction with methyl tert-butyl ether [113,117], ethyl tert-butyl ether [115], or n-butyl chloride [116].

However, solid phase extraction (SPE) seems promising. It yields clean chromatograms due to its capability of removing interferences from the matrix, leading to increased sensitivity, precision, and accuracy; it also presents short total analysis time and requires smaller sample volumes [33,114,118]. In fact, Mcintyre et al. [45] describe a fatal case report where peripheral blood, central blood, liver, vitreous, gastric content, and urine were used to screen for mitragynine by SPE (C_18_) followed by GC–MS. Using this approach the authors achieved limits of detection and limits of quantitation of 0.03 and 0.05 mg/L, respectively. Holler et al. [116] report the first publication of a death involving propylhexedrine and mitragynine. The authors determined mitragynine presence in urine and blood, after previous hydrolysis with β-glucuronidase and sulfatase. The extraction was performed with 3 mL of n-butyl chloride and the analysis was performed using a LC–MS/MS system. The authors achieved limits of detection and quantitation of 0.25 and 1 ng/mL respectively, and successfully applied the extraction technique to other specimens (liver, vitreous humor, kidney, heart, spleen, lung, and bile) in order to detect mitragynine. Heart was the only specimen in which the compound was not detected.

Bar adsorptive microextraction, using a modified N-vinylpyrrolidone polymer sorbent phase, combined with liquid desorption followed by HPLC–DAD, was used in human urine matrices (BAμE–LD/HPLC–DAD). This technique provided high selectivity for mitragynine, good performance, and it is easy to work-up and environmentally friendly [119].

Although most of these methods proved to be reliable for mitragynine analysis, they require complex sample preparations. They can also be too laborious, expensive, and not so easily accessible; therefore, further studies are required on this matter. One should keep in mind that so far kratom constituents are not detected by conventional drug screening tests (e.g., immunoassay tests).

Table 1 and Table 2 review the available literature (PubMed) about the analytical techniques (from 2010 to present) studied in human biological matrices and plant material.

## 8. Conclusions and Future Perspective

It is still very compelling for people to use substances that can enhance their abilities. Several NPS or so-called ‘designer drugs’ are usually used in non-medical scenarios as synthetic alternatives for illicit drugs of abuse. Kratom is an example of these new trended drugs. Its consumption is traditional in southern Thailand, and it was formerly consumed for the purposes of withstanding great heat and fatigue. Nowadays there are a wide amount of other reasons for consumption, such as opium substitution, to diminish opium addiction, or reduce pain from withdrawal symptoms. Unfortunately, kratom itself seems to cause dependence, therefore leading to withdrawal symptoms whenever people stop using it. It has also been reported to cause an increase in blood pressure, hepatotoxicity, nephrotoxicity, emphysema, over-inflation of the alveoli, and cytotoxicity to human neuronal cells and there are even several reports regarding fatalities after kratom consumption. This alone is a pretty good reason to pay more attention to this plant. It is important to develop simple, inexpensive, and effective methods for *Mitragyna speciosa* analysis so that more information about toxicity, interactions with other drugs, metabolic actions, and pharmacology can be understood. This becomes even clearer due to the ease of acquisition on the internet. The lack of a convenient test for mitragynine detection makes it a lot harder for authorities to detect kratom users, and therefore harder to provide health care for them. A small plant that was originally used traditionally in a particular region in Asia is now used worldwide with no need for medical prescription or supervision and for which a dependence treatment is yet to be known.

*Mitragyna speciosa* also seems to present interesting effects, namely antinociceptive, anti-inflammatory, gastrointestinal, anti-depressant, antioxidant, and anti-bacterial. Still, there is no sufficient evidence that supports its use for clinical purposes, and therefore further studies are required.

## Figures and Tables

**Table 1:** Analytical methods for the identification and/or quantification of *Mitragyna speciosa* in biological samples.

Compounds	Biological Sample (amount)	Analytical Technique	Internal Standard	Extraction Process	Linear Range (ng/mL)	LOD (ng/mL)	LOQ (ng/mL)	Recovery (%)	Reference
Mitragynine, 7-hydroxymitraginine, speciogynine, speciocilliatine, and paynantheine	Urine (1 mL)	LC–ESI–MS–QTOF	mitragynine-d_3_ 7-hydroxymitraginine-d_3_	Solid-phase extraction (PolyChrom ClinII cartridges)	2–500	0.25–1	0.5–1	96–63	[120]
Mitragynine, 5-desmethylmitragynine and 17-desmethyldihydro-Mitragynine, and 7-hidroxymitragynine	Urine (0.2 mL)	UHPLC–ESI/MS–MS and LC–ESI/MS–MS	Mitraphylline	Enzymatic hydrolysis and liquid-liquid extraction (*methyl* tert-*butyl ether*)	1–500	-	1.00	78–94	[113]
Mitragynine	Urine (1 mL)	HPLC–DAD	-	BAµE (N-vinylpyrrolidone polymer) and back-extraction with methanol/acetonitrile (1:1, *v*/*v*) under sonication	0.6–24	0.1	0.33	103	[119]
Mitragynine	Urine (1 mL)	LC–ESI–MS (QTRAP)	methyltestosterone	Enzymatic hydrolysis and liquid-liquid extraction (ethyl tert-butyl ether)	0.25–1.5	0.2	0.25	83	[115]
Mitragynine	Peripheral blood, central blood, liver, vitreous, gastric content, and urine (1 mL)	GC–MS (EI)	mitragynine-d_3_	Solid-phase extraction (Trace-J cartridges)	50–1000	30	50	-	[45]
Mitragynine, paynantheine, speciogynine, speciociliatine, 16-carboxy-mitragynine, 9-O-demethyl-mitragynine, and 9-O-demethyl-16-carboxy-mitragynine	Urine (3 mL)	GC–MS (EI)	-	Enzymatic hydrolysis and solid-phase extraction (HCX cartridge)	-	100	-	-	[110]
Mitragynine	Urine (2 mL)	LC–ESI/MS–MS	Ajmalicine	Liquid extraction; liquid-liquid extraction (*Methyl tert-butyl ether*)	0.01–5	0.02	0.1	81	[117]
Mitragynine	Blood and urine (1 mL), tissues (liver, kidney, heart, spleen, lung −1g), bile, and vitreous humor	LC–ESI/MS–MS	Proadifen	Enzymatic hydrolysis and liquid-liquid extraction (n-butyl chloride)	1–10	0.25	1	103	[116]
Mitragynine	Rat serum (0.1 mL)	HPLC–UV LC–ESI–MS	Acenapthene	Liquid–liquid extraction (diethyl ether)	100–10,000	30	100	85–84	[121]
Mitragynine	Rat plasma (0.1 mL)	HPLC–UV	Mefloquine	Solid-phase extraction (MCX Oasis cartridges)	50–1000	25	50	96–98	[33]
Mitragynine	Rat and human urine (1 mL)	HPLC–DAD	-	Solid-phase extraction (Oasis^®^ HLB cartridge)	100–10,000	-	100	93–101	[118]
Mitragynine and metabolites	Rat and human urine (1 mL)	LC–ESI.LIT and LC–ESI.Orbitrap MS	-	Enzymatic hydrolysis and solid-phase extraction (Isolute Confirm HCX and Isolute Confirm C18 cartridges)	-	-	-	-	[114]

BAµE: Bar adsorptive microextraction; DAD: Diode-array detection; EI: Electron ionization mode; ESI: Electrospray ionization; GC: Gas chromatography; HPLC: High-performance liquid chromatography; HPLC–UV: High-pressure liquid chromatography with ultraviolet detector; LC: Liquid chromatography; LD: Liquid desorption; LIT: Linear ion trap; MS: Mass spectrometry; LOQ: Limit of detection; LOQ: Limit of quantitation; MS/MS: Tandem mass spectrometry; MTBE: Methyl t-butyl ether; RP–HPLC: Reverse-phase high performance liquid chromatography; SIM: Selective ion mode; TBME: t-Butyl methyl ether; TOF: Time of flight; UHPLC: Ultra-high-performance liquid chromatography.

**Table 2:** Analytical methods for the identification and/or quantification of *Mitragyna speciosa* in leaves and plant material.

Compounds	Sample (Amount)	Analytical Technique	Extraction	LOD	LOQ	Recovery (%)	Reference
Several secondary metabolites	Mature leaves (100 mg)	LC–ESI–TOF–MS	Ice cold methanol	-	-	-	[31]
Mitragynine	Dried leaves (1.13 kg)	icELISA and HPLC–DAD	Methanol maceration, acid-base extraction, and silica gel column chromatography	32.47 μg/mL	-	-	[109]
Mitragynine	Leaves (5 kg)	HPLC–DAD	Methanol maceration and liquid extraction (chloroform)	0.25 μg/mL	0.5 μg/mL	95–101	[107]
	Ketum drink (1 mL)		Direct injection				
Mitragynine 7-OH mitragynine	Raw materials and powdered extracts (100 mg) and capsules	HPLC–UV	For dry test materials (0.5 M acetic acid in 70% methanol) For beverages (dilution with methanol)	0.002% (*w*/*w*)	0.006% (*w*/*w*)	94–95	[111]
	Liquid finished products and/or beverages			0.2 μg/mL	0.6 μg/mL		
7-hidroxymitragynine	Raw materials, powdered extracts, and capsules			0.004% (*w*/*w*)	0.011% (*w*/*w*)	96–99	
	Liquid finished products and/or beverages			0.4 μg/mL	1.1 μg/mL		
Mitragynine	Ketum cocktail	HPLC–DAD	Freeze drying and reconstitution with methanol:water (80:20, *v*/*v*)	1.000 μg/mL	3.000 μg/mL	95	[122]
Mitragynine	Dried plant material (2 g)	TLC and HPLC–UV	Ethanol	1 μg /mL	-	-	[106]
Mitragynine	Kratom (powder or ground leaves material (100 mg); ¼ tea-spoon, liquid: 250 μL, capsule: 1)	IMS and LC–MS/MS	Methanol and ultrasonic bath sonication	0.5 ng/μL	-	-	[108]
				2000 μg/mL	6000 μg/mL		
Mitragynine, 7-hydroxymitragynine, and mitraphylline (stereoisomers, mitraciliatine, speciogynine, speciociliatine)	Plant material as fresh leaves (1.0 cm × 0.5 cm)	HR–DART–MS	Ethanol	-	-	-	[32]

DAD: Diode-array detection; DART: Direct analysis in real time; HPLC: High-performance liquid chromatography; HPLC–UV: High-pressure liquid chromatography with ultraviolet detector; HR: High resolution; icELISA: Indirect competitive enzyme-linked immunosorbent assay; IMS: Ion mobility spectrometry; LC: Liquid chromatography; LDA: Linear discriminant analysis; LOQ: Limit of detection; LOQ: Limit of quantitation; MS: Mass spectrometry; MS/MS: Tandem mass spectrometry; TLC: Thin layer chromatography; TOF: Time of flight.
